# Quantitative Analysis of Isoflavones from *Fabaceae* Species and Their Chemopreventive Potential on Breast Cancer Cells

**DOI:** 10.3390/molecules30112379

**Published:** 2025-05-29

**Authors:** Wojciech Paździora, Karolina Grabowska, Paweł Zagrodzki, Paweł Paśko, Ewelina Prochownik, Irma Podolak, Agnieszka Galanty

**Affiliations:** 1 Doctoral School of Medical and Health Sciences, Jagiellonian University Medical College, 16 Łazarza Str., 31-530 Cracow, Poland; wojciech.pazdziora@doctoral.uj.edu.pl; 2 Department of Pharmacognosy, Jagiellonian University Medical College, Medyczna 9, 30-688 Kraków, Poland; karolina1.grabowska@uj.edu.pl (K.G.); irma.podolak@uj.edu.pl (I.P.); 3 Department of Food Chemistry and Nutrition, Jagiellonian University Medical College, Medyczna 9, 30-688 Kraków, Poland; pawel.zagrodzki@uj.edu.pl (P.Z.); p.pasko@uj.edu.pl (P.P.); ewelina.gajdzik@uj.edu.pl (E.P.)

**Keywords:** *Fabaceae*, isoflavones, *Trifolium*, extraction optimization, cytotoxic, breast cancer

## Abstract

The *Fabaceae* family is known for the presence of isoflavones—phytoestrogens with potential chemopreventive effects against hormone-dependent cancers. This study aimed to optimize isoflavones extraction using a fractional factorial design and to quantitatively and qualitatively analyze 32 *Fabaceae* species native to Polish flora by HPLC-UV-VIS to indicate new, rich plant sources of isoflavones. The optimal extraction method was a 60 min reflux with 50% methanol and a plant material-to-solvent ratio of 1:125. The highest isoflavone levels were found in *Trifolium medium* (26.70 mg/g d.m.), *Genista tinctoria* (19.65 mg/g d.m.), and *Trifolium pratense* (12.56 mg/g d.m.). The obtained extracts were further evaluated for cytotoxic and antiproliferative activity against MCF7 and MDA-MB-231 human breast cancer cells. *Genista tinctoria* showed the highest cytotoxicity against MCF7, while *Cytisus scoparius* and *Ononis arvensis* were most effective against MDA-MB-231 at a dose of 500 µg/mL. The extracts were also characterized by varied, potent antioxidant properties, important in chemoprevention. A strong correlation was observed between isoflavone content and cytotoxic and antiproliferative activity exclusively in the estrogen receptor-positive MCF7 cell line. Importantly, the tested extracts demonstrated no toxic effects on normal human liver (HepG2), thyroid (Nthy-ori 3-1), or breast (MCF10A) cells, indicating a favorable safety profile.

## 1. Introduction

Plants from the *Fabaceae* family are an attractive subject of scientific research due to their wide distribution in nature and health-promoting potential, resulting from the presence of polyphenolic compounds [1]. Among them, isoflavones, also called phytoestrogens, are suggested to be the chemotaxonomic markers of the *Fabaceae* family [2]. A characteristic structural feature of isoflavones is the similarity to estrogens, female sex hormones, resulting in the ability to bind to the estrogen receptor in the cell nucleus. The presence of hydroxyl groups in the isoflavone structure, especially in the 5- and 7-positions of the ring, determines the biological activity towards this receptor, due to the structural similarity to 17-β-estradiol [3]. This may be of great importance in terms of the development of hormone-dependent cancers, especially those with the expression of estrogen receptors (e.g., breast, prostate, ovarian cancers), because these compounds may compete with 17-β-estradiol for the binding site of estrogen receptors [4,5]. Literature data from the in vitro studies indicate epigenetic mechanisms of isoflavones, including DNA methylation, histone modification and expression of non-coding RNA, and thus modulation of pro-cancer gene expression [6]. Genistein has been shown to reduce the levels of DNA methyltransferases (DNMT1, DNMT3a, and DNMT3b) in breast cancer cell lines (MCF7 and MDA-MB-231) [7], while genistein and daidzein reduced the methylation of genes involved in DNA repair (BRCA1, GSTP1, and EPHB2) in DU-145 and PC-3 prostate cancer cell lines [8]. Thus, the search for novel, rich plant sources of isoflavones, in terms of their potential use in chemoprevention, is still of high importance.

Breast cancer remains one of the most commonly diagnosed malignancies and a leading cause of cancer-related mortality among women worldwide. Despite significant advancements in early detection and therapeutic approaches, the rising global incidence underscores the urgent need for effective, preventive solutions [9]. Among chemopreventive agents, plant-derived compounds have garnered increasing scientific attention due to their multi-targeted biological activity and relatively favorable safety profiles. Natural compounds, including isoflavones found in *Fabaceae* species, represent a promising group of chemopreventive agents due to their ability to modulate hormone-dependent pathways, exert antioxidant effects, and influence epigenetic regulation. Their potential to prevent or delay tumor development, especially in high-risk groups, highlights the need for continued search for novel, isoflavones-rich plant-based therapeutics in comprehensive breast cancer prevention strategies [10,11].

The *Fabaceae* family includes several species that are exceptionally rich in isoflavones and are also used in medicine and pharmacy in the form of over-the-counter drugs and dietary supplements. These include soybeans, with the content of isoflavones reaching as much as 1932.4 μg/g dry weight [12], the aboveground parts of *Genista tinctoria* with isoflavones up to 58.7 mg/g d.m. [13], the roots of *Ononis* sp., containing up to 3.63 g/100 g of isoflavones [14], or *Trifolium pratense*, the flowers of which are used during menopause. Despite a large number of qualitative and quantitative analyses of isoflavones in different *Fabaceae* species, what was recently reviewed by our research group [15], there are still some gaps to be filled, including a number of species still analyzed only qualitatively [16,17]. Moreover, the isoflavones content in *Fabaceae* species native to Poland was described mainly for several *Trifolium* species [18], which represents another gap that needs to be addressed.

Therefore, the aim of our study was to conduct a comprehensive screening of isoflavone content in *Fabaceae* plants native to Poland to identify new sources of these compounds and assess their chemopreventive potential against breast cancer cells. To achieve these goals, we applied a fractional factorial plan to optimize the extraction conditions of isoflavones, which were subsequently used for preparing the extracts for the qualitative and quantitative HPLC analysis. Next, the chemopreventive potential, including cytotoxic, antiproliferative, and antioxidant activity of the extracts with the determined amount of isoflavones, was evaluated. Finally, a safety and selectivity assessment of the extracts was performed.

## 2. Results and Discussion

### 2.1. Extraction Optimization

The first step of the experiment included the optimization of the extraction process to preselect the most effective extraction conditions for obtaining isoflavones. The optimization was performed for the Trifolium pratense herb, known for its high amount and diversity of isoflavones [15]. For this purpose, we focused on two different extraction techniques, and within each of these techniques, the influence of three selected parameters on the extraction efficiency was examined (see Section 3.2. for details). Then, the best scores from each extraction type were indicated to finally choose the optimal extraction process. The choice of methods and parameters was determined by literature data. The most commonly used extraction methods of isoflavones are heat-reflux extraction [19,20] and ultrasound-assisted extraction [14,18,19,21,22,23]. Most studies used methanol as a solvent, in various concentrations ranging from 50 to 100% [24,25].

The results obtained for the optimization process are presented in Table 1, as the isoflavones sum. The isoflavones sum was the highest in set 9 (i.e., 120 min extraction under heating reflux with 100% methanol solution, plant material-to-solvent ratio of 1:125). The values obtained in set 9 differed significantly from all other sets (*p* < 0.05), except for set 4, which yielded the second-highest result (i.e., 60 min extraction under heating reflux with 50% methanol solution, plant material-to-solvent ratio of 1:125). Interestingly, the two sets with the highest scores differed with the extraction time (120 vs. 60 min) and solvent concentration (100 vs. 50%). However, we decided to choose set 4 for further quantitative analysis, due to its more environmentally friendly and cost-effective conditions (lower methanol concentration and shorter extraction time) compared to set 9.

### 2.2. Qualitative and Quantitative Analysis of Fabaceae Plants

**Figure 1:**
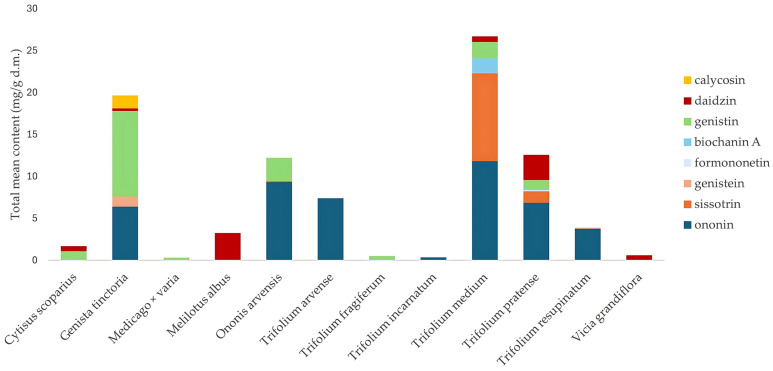
Variability of the total mean content (mg/g d.m.) of all isoflavones (with specified content of individual compounds, where possible), determined by HPLC, within the tested species of the *Fabaceae* family.

In our search for new plant sources of isoflavones, we focused on the species growing in Poland, relatively easily accessible in the natural environment. The inclusion criteria covered herbaceous plants, occurring in the wild flora. The exclusion criteria included trees, species cultivated for ornamental or edible purposes, or species with the status of legally protected plants. In total, 32 plant species were collected and examined (see Section 3.5 for details). The isoflavone content, determined based on our available reference substance database, could be quantified in only 12 species of the collected plants. The results of the quantitative analysis are presented in Figure 1.

*Trifolium* was the most widely represented genus in the study, with ten species included (*Trifolium arvense*, *Trifolium campestre*, *Trifolium fragiferum*, *Trifolium hybridum*, *Trifolium incarnatum*, *Trifolium medium*, *Trifolium montanum*, *Trifolium pratense*, *Trifolium repens*, and *Trifolium resupinatum*). The highest isoflavones content was obtained in the extracts from *Trifolium pratense* and *Trifolium medium*, with 26.7 and 12.56 mg/g d.m. of their sum, respectively. In the remaining *Trifolium* species, the content was noticeably lower or not detected. Additionally, *Trifolium pratense* and *Trifolium medium* were characterized by the highest qualitative diversity of isoflavones, with seven compounds found (Table 2).

In *Trifolium pratense*, ononin and daidzin predominated, while in *Trifolium medium*, the dominant isoflavones were ononin, and sissotrin. The results for isoflavone composition in *Trifolium medium* contrast with those reported by Zgórka et al., where biochanin A-7-O-glucoside-6-O-malonate (20.63 mg/g d.m.) and formononetin-7-O-glucoside-6-O-malonate (7.31 mg/g d.m.) were the predominant compounds. Notably, these two compounds accounted for as much as 54.3% of the total isoflavone mass in the plant, whereas aglycones represented only 16.6% [26]. Several methodological and environmental differences between the two studies may account for these discrepancies. First, the plant material in the study by Zgórka et al. [26] was collected in eastern Poland, whereas our samples were harvested in a different region of the country (south Poland). Second, the drying conditions of plant material (an elevated temperature of 35 °C vs. room temperature in our study) also may result in the discrepancies, potentially affecting the thermal stability of glycosidic and malonylated isoflavones. Furthermore, our extract was not lyophilized, in contrast to Zgórka et al. [26], which can also influence the isoflavones profile. Moreover, the extraction procedure differed significantly. Zgórka et al. [26] applied ultrasound-assisted extraction using 50% ethanol and repeated the procedure three times, while our study used a single-step heat-reflux extraction with methanol under a condenser, which may selectively favor certain isoflavones over others due to heat exposure and reduced extraction time. On the other hand, Butkute et al. found only three isoflavones (daidzein, formononetin, and genistein) in the aerial parts of Trifolium pratense, with lower content than in our study [27], while Hloucalová et al. detected seven isoflavones, but the total content was lower than what we obtained [28]. In the extract from *Trifolium arvense*, ononin was the only identified isoflavone (7.38 ± 0.09 mg/g d.m.). *Trifolium fragiferum* contained small amounts of genistin (0.50 ± 0.00 mg/g d.m.), but the amount of the compound was much higher than previously reported by Visnevschi-Necrasov et al. (13.2 mg/kg d.m.) [29]. The same authors determined 11 isoflavones in the species but only in trace amounts. In *Trifolium incarnatum*, only ononin was identified, although Zgórka et al. [17] and Vetter [30] determined several other compounds. Ononin and genistein were determined in *Trifolium resupinatum* (3.76 ± 0.04 and 0.11 ± 0.02 mg/g d.m., respectively), but in significantly lower amounts than the values obtained by other authors [28,29].

*Ononis arvensis* is a rarely studied species, in contrast to the more extensively investigated *Ononis spinosa*. Gampe et al. showed a wide range of isoflavones (e.g., pseudobaptigenin 7-O-glucoside, formononetin 7-O-glucoside, onogenin 7-O-glucoside, pseudobaptigenin, formononetin) in *Ononis arvensis*, but they did not reveal the presence of ononin [14]. Thus, our quantitative analysis was performed for the first time on this species and indicated that the plant is a rich source of ononin (9.34 ± 0.17 mg/g d.m.), which also refers to its Latin name. For comparison, the content of this compound in *Ononis spinosa* was 1.76 mg/g d.m. [31]. Other isoflavones noted in our study for *Ononis arvensis* were sissotrin (0.08 ± 0.01 mg/g d.m.), and genistin (2.80 ± 0.09 mg/g d.m.).

*Genista tinctoria* was the richest source of genistin among the species tested, the content of which was as much as 10.20 ± 0.22 mg/g d.m. The extract also contained ononin (6.41 ± 0.08 mg/g d.m.), genistein (1.18 ± 0.09 mg/g d.m.), daidzin (0.33 ± 0.01 mg/g d.m.), and calycosin (1.54 ± 0.03 mg/g d.m.). The amount of genistein is much higher than that obtained by Vlase et al. (0.72 mg/g d.m.) [32]. There are several quantitative studies of this species, but the data are presented as the isoflavones content in the extract, which makes it impossible to compare. For example, Rigano et al. determined the amount of genistein to be 535.21 mg/g extract [33], while Hangau et al. determined 73.77 μg/mL of the extract [20]. The authors also mentioned the presence of other isoflavones like formononetin, orobol, and isoprunetin.

The extract from *Cytisus scoparius* contained daidzin (0.60 ± 0.03 mg/g d.m.) and also genistin (1.08 ± 0.02 mg/g d.m.). These amounts are much higher than those determined by Cunha et al. in the extracts from leaves and stems, with 21 mg/kg d.m. for daidzin [34].

Our study is the first to reveal the presence of daidzin in the extract from *Melilotus albus*, with the content of 3.23 ± 0.11 mg/g d.m. This plant is the richest source of this compound among all the tested plants. Furthermore, there is no literature data on the qualitative isoflavone composition of *Melilotus albus*.

The genus *Medicago* was represented in our study by three species (*Medicago falcata*, *Medicago lupulina*, and *Medicago x varia*). The only compound quantitatively determined was genistin (0.32 ± 0.01 mg/g d.m.) in *Medicago x varia*, which is a hybrid of *Medicago sativa* and *Medicago falcata*. The species has not been previously subjected to qualitative/quantitative analysis. In *Medicago falcata*, no isoflavones were identified, which is consistent with literature data.

The genus *Vicia* was represented in our study by five species (*Vicia angustifolia*, *Vicia grandiflora*, *Vicia hirsuta*, *Vicia sepium*, and *Vicia villosa*). In the literature, the only data on isoflavones content are available for *Vicia faba*, with daidzein and genistein in the range 1.03–92 μg/g d.m. [35,36]. Therefore, we demonstrated for the first time the presence of daidzin in *Vicia grandiflora*. What is more, the content of daidzin in *Vicia grandiflora* (0.58 ± 0.03 mg/g d.m.) was noticeably higher than the reported levels in the previously mentioned species.

**Figure 2:**
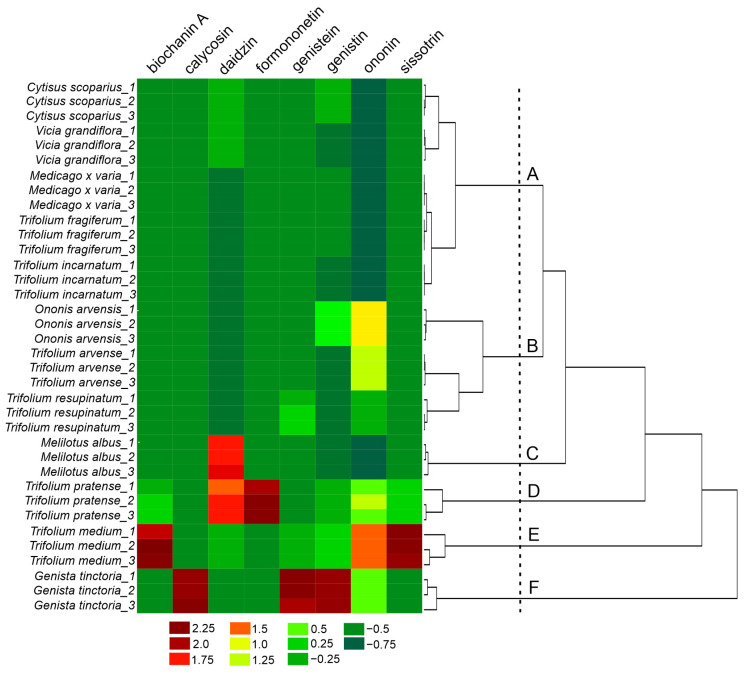
The heatmap and dendrogram to visualize the clustering of *Fabaceae* species based on the contents (mg/g d.m.) of isoflavones (biochanin A, calycosin, daidzin, formononetin, genistein, genistin, ononin, sissotrin). Clusters were marked with subsequent letters: A–F; method of grouping: Ward’s method.

To better highlight the similarities and/or differences in isoflavones content between the analysed *Fabaceae* species, hierarchical cluster analysis (HCA) was performed using Ward’s method and Euclidean distance, resulting in the formation of six clusters: A–F (Figure 2). Clusters A and B contained species in which the presence of biochanin A, calycosin, and formononetin was not detected. Within cluster A, the greatest similarity in daidzin content was observed between *Cytisus scoparius* and *Vicia grandiflora*, while the highest similarity in genistin content was noted between *Trifolium fragiferum* and *Medicago x varia*. In contrast to cluster B, none of the species in cluster A contained genistein or sissotrin. Overall, the species grouped in cluster A exhibited markedly lower levels of isoflavones than those in the remaining clusters. In turn, cluster B included three species (*Ononis arvense*, *Trifolium arvense*, *Trifolium resupinatum*) characterized by significant content of ononin but the absence of daidzin. The remaining clusters (C–F) formed separate, single-element (single-species) groups, as each of the analyzed plant species exhibited a unique pattern of isoflavone presence and content. Cluster C (*Melilotus albus*) was characterized by a high content of daidzin, with a simultaneous absence of other isoflavones. Conversely, cluster D (*Trifolium pratense*) was the only one in which formononetin was detected. Cluster E (*Trifolium medium*) was distinguished by high levels of sissotrin, ononin, and biochanin A, compared to the other *Fabaceae* species included in the study. Cluster F (*Genista tinctoria*), similarly to clusters B, C, and D, also showed a significant content of ononin. However, unlike the other clusters, it exhibited the highest level of genistin and was the only one to contain calycosin.

### 2.3. Assessment of Chemopreventive Properties of the Extracts

In the next step of our study, the impact of the examined plant extracts—characterized by varying isoflavone content—on viability and proliferation of human breast adenocarcinoma cells was evaluated. The selection of the cell lines was intended to reflect the heterogeneous nature of the tumor, usually consisting of the cells differing in their metastatic potential or receptor exposure [37]. Thus, in consideration of the estrogenic properties of isoflavones, estrogen receptor positive MCF7 cells were used and compared with estrogen receptor negative and highly metastatic MDA-MB-231 cells.

#### 2.3.1. Effect on the Viability of Cancer Cells

**Figure 3:**
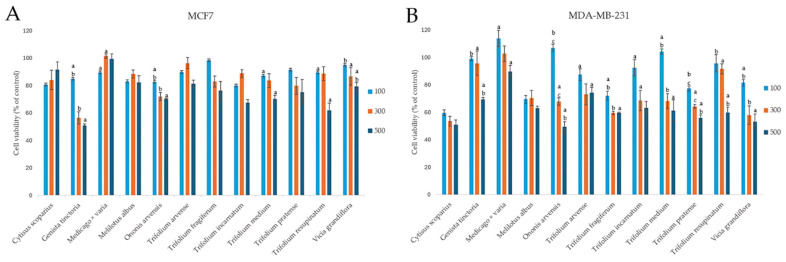
Cytotoxic effect of extracts from selected Fabaceae species on breast cancer MCF7 (**A**) and MDA-MB-231 (**B**) cells. Cells were treated with 25–500 µg/mL of plant extracts (n = 3) for 48 h. The figure presents results for concentrations ranging from 100 to 500 µg/mL. Significant differences between extract doses within a particular species are indicated by different lowercase letters (*p* < 0.05; ANOVA, Tukey’s post hoc test).

The cytotoxic potential of the examined plants from the *Fabaceae* family was tested within the wide concentration range, from 25 to 500 µg/mL. However, as no effect was observed at the concentrations below 100 µg/mL, the obtained results are presented for three highest tested concentrations in Figure 3. In general, the examined extracts revealed a rather moderate cytotoxic effect, according to the criteria of the National Cancer Institute and Geran protocol for plant extracts [38], with highly invasive MDA-MB-231 cells exhibiting greater susceptibility to the tested extracts compared to the estrogen-dependent MCF7 cells. The strongest decrease in MCF7 cells viability was noted for the extract from *Genista tinctoria*, with 50.74 ± 1.44% viable cells at the highest tested concentration, while for MDA-MB-231 cells, the strongest impact on cell viability was observed for the extracts from *Ononis arvensis* and *Cytisus scoparius* (49.42 ± 3.80 and 50.85 ± 3.51%, respectively, at the highest concentration tested). The weakest effect on the viability of both cell lines was observed for the extract from *Medicago x varia*.

Our results for the *Trifolium pratense* extract are in contrast with Zgórka et al. [26], who reported approximately twofold greater decrease in MCF7 and MDA-MB-231 cells’ viability in an MTT assay after 48 h of exposure to 500 and 1000 µg/mL of extract, with MCF7 cells being more sensitive. On the other hand, *Trifolium pratense* extract did not demonstrate cytotoxic activity against MCF7 cells in the study by Booth et al. [39]. The results for the cytotoxic activity of other *Fabaceae* species examined in our study, i.e., *Cytisus scoparius*, *Genista tinctoria*, *Medicago x varia*, *Melilotus albus*, *Ononis arvensis*, *Trifolium arvense*, *Trifolium fragiferum*, *Trifolium incarnatum*, *Trifolium medium*, *Trifolium resupinatum*, and *Vicia grandiflora*, have been reported for the first time. Thus, we can only compare the obtained effects with other species from the genus. For example, the IC_50_ values of *Ononis hirta* extracts against MCF7 cells were 44.58 ± 1.42 μg/mL, 72.06 ± 2.79 μg/mL, and 27.96 ± 0.54 μg/mL for chloroform, n-hexane, and methanol extracts, respectively. Similarly, IC_50_ values for *Ononis sicula* were reported as 66.02 ± 1.58 μg/mL and 114.11 ± 2.42 μg/mL for chloroform and n-hexane extracts, respectively [40]. A weak cytotoxic effect against MCF7 cells was also observed by Asadi-Samani et al. [41], where the IC_50_ value for *Medicago sativa* extract was reported to be <300 µg/mL. In a separate screening study evaluating the cytotoxic activity of 76 plant species from various families against MCF7 cells, after 72 h of exposure, survival rates were reported as 80.30 ± 6.31%, 90.01 ± 4.68%, and 95.79 ± 5.76% for *Melilotus indicus*, *Ononis natrix*, and *Trifolium purpureum*, respectively [42]. Another study on *Ononis natrix* demonstrated weaker cytotoxic activity (IC_50_ = 28.75 ± 2.5 μg/mL) in comparison to tamoxifen (IC_50_ = 10.5 ± 1.7 μg/mL) against estrogen receptor-negative MDA-MB-231 cells [43]. The infusion and hydroxyethanolic extracts of *Genista tridentata* revealed varied cytotoxic effects on MCF7 cells, with GI_50_ values of 129.1 ± 6.3 μg/mL and 146.8 ± 6.5 µg/mL, respectively [44].

#### 2.3.2. Effect on the Proliferation of Cancer Cells

**Figure 4:**
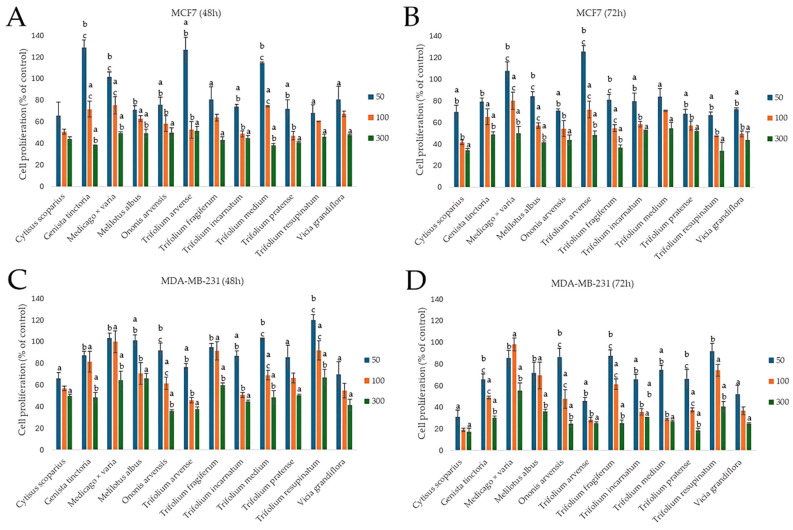
Antiproliferative effect of the extracts of selected Fabaceae species on breast cancer MCF7 (**A**,**B**) and MDA-MB-231 (**C**,**D**) cells. Cells were treated with 50, 100, and 300 µg/mL of plant extracts (n = 3) for 48 h or 72 h. Significant differences between extract doses within a particular species are indicated by different lowercase letters (*p* < 0.05; ANOVA, Tukey’s post hoc test).

Apart from the impact on cancer cell viability, chemopreventive agents should also affect other aspects of cellular functioning, e.g., the proliferation rate, the decrease of which may lead to the reduced growth of the tumor. On the other hand, it is known that isoflavones can stimulate the proliferation of breast cancer cells, especially those with the estrogenic receptor exposed [45]. Thus, we decided to verify the antiproliferative potential of the examined extracts, assessed at two time points: 48 and 72 h. Cells were treated with subcytotoxic doses of 50, 100, and 300 µg/mL of each extract. The results showed both dose- and time-dependent effects, with interesting differences between the two cell lines. Overall, MDA-MB-231 cells showed greater sensitivity to the tested extracts than MCF7 cells, especially after 72 h. This observation is of great importance, considering the aggressive, refractory nature of MDA-MB-231 triple-negative breast cancer, which may suggest a selective susceptibility to some phytochemicals present in these extracts. In both cell lines and time points, the extracts from *Cytisus scoparius*, *Genista tinctoria*, *Trifolium arvense*, and *Ononis arvensis* appeared as the most effective. In both cell lines, extending the treatment from 48 to 72 h generally enhanced the antiproliferative effects, as observed for *Cytisus scoparius*, *Genista tinctoria*, and *Ononis arvensis*. Interestingly, some extracts in lower concentrations seemed to slightly stimulate cell proliferation, especially after 48 h of incubation in estrogen-dependent MCF7 cells. This was particularly characteristic for plants with high isoflavones sum content, as observed at 50 µg/mL concentration for *Genista tinctoria*, *Trifolium arvense*, and *Trifolium medium* (117.86 ± 22.40%, 126.58 ± 11.71%, 114.60 ± 0.94% of control, respectively) (Figure 4).

Our results are difficult to compare with those of other authors, as most of the reported studies focused mainly on estrogenic or cytotoxic effects, omitting the aspect of the influence on proliferation as an element of chemoprevention. Ethyl acetate extract from *Cytisus villosus* shows a stronger antiproliferative effect on MCF7 cells than the aqueous extract [46]. The aqueous extract of *Ononis spinosa* inhibited cell proliferation of the MD-MBA-231 line by 30.11 ± 0.85% or 49.05 ± 1.47% after 48 h or 72 h, respectively, at the highest concentration (1 g/L), but the authors did not determine the content of isoflavones in the extract [47]. The extract from the same species showed antiproliferative activity against the estrogen-dependent MCF7 line, and the IC_50_ was 101.28 ± 8.29 μg/mL [48]. To our best knowledge, we reported for the first time the antiproliferative effect of the examined *Fabaceae* species.

#### 2.3.3. Antioxidant Potential

Isoflavones, classified as polyphenolic compounds, possess not only phytoestrogenic properties but also significant antioxidant potential [49]. Oxidative stress is recognized as a critical factor in the initiation and progression of carcinogenesis [50]. Moreover, certain antioxidant agents have demonstrated the ability to mitigate the toxic effects of chemotherapy and radiotherapy on normal, healthy tissues [51]. Therefore, to complete the evaluation of the chemopreventive properties of the extracts tested, we determined their antioxidant potential, and the results are presented in Table 3. In both the DPPH and FRAP assays, the extract from *Trifolium arvense* achieved the highest values (182.4 ± 9.3 μM TEAC/g d.m. and 248.8 ± 8.6 μM Fe^2+^/g d.m., respectively). However, these values were not significantly different from those obtained for the extract from *Cytisus scoparius*, with the second highest score. In a similar study, in the DPPH and ABTS tests, *Cytisus villosus* extracts showed remarkable antioxidant activity, with IC_50_ values comparable to or even better than standard antioxidants such as BHT and ascorbic acid (e.g., DPPH IC_50_ 3.94 μg/mL for H_2_O extract vs. 4.15 μg/mL for BHT) [52]. In contrast, the extract of *Cytisus scoparius* in our study showed relatively high antioxidant capacity in DPPH and FRAP assays, with values of 178.07 ± 5.89 μM TEAC/g d.m. and 232.2 ± 3.68 μM Fe^2+^/g d.m., respectively. These results indicate strong reducing capacities, although direct comparison with IC_50_ values from other species is limited due to different units and methodologies.

### 2.4. Safety Assessment

Safety assessment of the candidates for chemopreventive agents is crucial during the evaluation of their effectiveness. Thus, the next part of our experiment was to verify the potential hepatotoxic and goitrogenic effects and also the impact on non-cancerous breast cells of the examined extracts, achieved by the use of human hepatocellular carcinoma HepG2, thyroid follicular epithelial Nthy-ori 3-1, and breast epithelial MCF10A cells, respectively. Despite their cancer origin, HepG2 cells reveal phenotypic characteristics similar to normal hepatocytes and are often used for assessing hepatotoxicity [53]. We have also included normal thyroid cells as isoflavones due to their estrogenic properties, as they may stimulate the excessive growth of thyroid gland, revealing goitrogenic effect [54]. To verify the safety but also the selectivity of the examined extracts, non-cancerous breast epithelial cells were also included in the study. The obtained results showed no hepatotoxic effects of the extracts. The lowest cell viability was approximately 61.40 ± 6.65% at the highest tested concentration of *Ononis arvensis* extract, in comparison to doxorubicin (IC_50_ = 1.0 μg/mL, 24 h). The same extract exhibited a similar effect on thyroid cells, with a viability of 60.24 ± 4.74%. *Trifolium incarnatum*, *Trifolium resupinatum*, and *Cytisus scoparius* extracts, at the highest tested dose of 500 μg/mL, demonstrated moderate antiproliferative effects on normal thyroid cells, with cell proliferation decreased to 62.14 ± 2.29%, 62.47 ± 0.79%, and 63.74 ± 0.40%, respectively. The examined extracts were selective and did not affect the viability of non-cancerous breast epithelial cells MCF10A (cell viability did not decrease below 80% of control). The overall results indicate that the extracts are of high safety; however, this should be verified in future animal studies.

### 2.5. Influence of Isoflavone Content on the Results of In Vitro Studies

**Figure 5:**
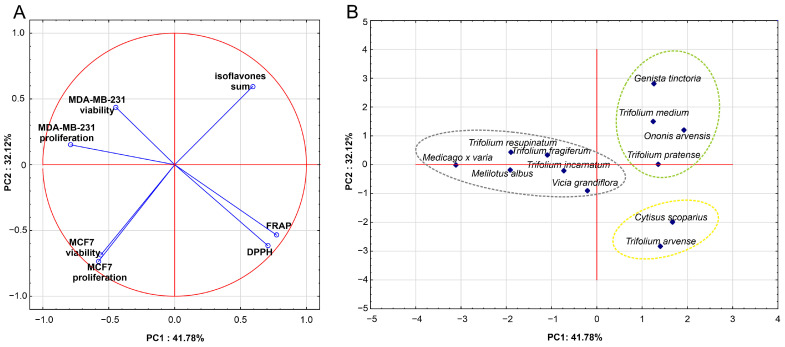
PCA correlation circle displaying the variables as vectors in a space created by the two principal components (PC1 and PC2) (**A**); PCA score scatterplot for analyzed *Fabaceae* species in the space defined by PC1 and PC2 (**B**).

To assess the similarities between the analyzed *Fabaceae* species based on isoflavones content (sum of isoflavones) and their biological activity, principal component analysis (PCA) and hierarchical cluster analysis (HCA) were conducted. In the constructed PCA model, the plant species served as cases, while the measured parameters (proliferation and viability of cell lines after 48 h of incubation, antioxidant activity, sum of isoflavones) were used as variables. The PCA model had two significant components, with eigenvalues higher than 1 (2.92 and 2.25, respectively), and explained 74% of the variance in the dataset. The first principal component was most strongly correlated (positively) with features such as antioxidant potential (FRAP, DPPH) and the impact on the proliferation of hormone-negative (MDA-MB-231) cell line (negatively correlated). In turn, the second component was mainly negatively correlated to the viability and proliferation of the hormone-positive (MCF7) cell line, followed by antioxidant potential and isoflavone content, with slightly lower correlations (Figure 5A). The characteristics of the model are presented in Table 4.

The correlation circle for the first two principal components (Figure 5A) illustrates that the content of isoflavones in the analyzed species did not correlate with their antioxidant activity (DPPH) and had a weak positive correlation with FRAP (r = 0.24). These data suggest that other compounds, including other polyphenols present in the plant species, are probably responsible for the observed antioxidant potential. In turn, the PCA model showed moderate negative correlations between the antioxidant activity of the extracts (DPPH, FRAP) and the viability (r = −0.39; r = −0.40, respectively) and proliferation (r = −0.53; r= −0.56, respectively) of the estrogen-negative MDA-MB-231 cells (Figure 5A), while no correlation was observed between the sum of isoflavones and the effect on the viability of these cells. In contrast, the PCA model indicated moderate negative correlation between isoflavones content and cell viability (r = −0.57), as well as strong negative correlation with proliferation of the estrogen-positive MCF7 cell line (r = −0.69) (Figure 5A). This result suggests that higher isoflavones content in the examined extracts strongly affected the viability and proliferation of hormone-positive MCF7 cells.

**Figure 6:**
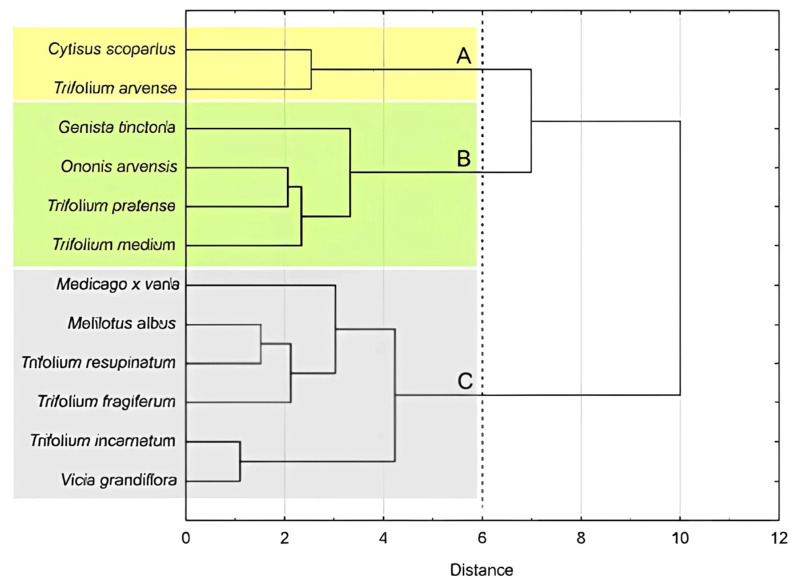
Dendrogram of similarity among investigated *Fabaceae* species. Clusters A–C identified using Mojena’s rule (dashed line).

The projection of *Fabaceae* species into the space defined by the first two principal components of PCA (Figure 5B) revealed three distinct groups of the *Fabaceae* species, each sharing similar overall profile of the measured features. The same three clusters (A, B, C) of Fabaceae species were confirmed by the classification based on the HCA method (Figure 6).

Cluster A included two species (*Cytisus scoparius*, *Trifolium arvense*) characterized by high antioxidant activity and moderate isoflavone content. The overall inhibitory effect on MDA-MB-231 cells’ proliferation was comparable to the species within cluster B but stronger than that observed within cluster C, while the inhibitory effect on MCF7 cell proliferation was weaker than in cluster B. Group B (cluster B) included species (*Genista tinctoria*, *Ononis arvensis*, *Trifolium pratense*, *Trifolium medium*) with the highest content of isoflavones as well as the highest inhibiting activity on proliferation and viability of MCF7 cells. Species of this cluster were characterized by comparable activity to cluster A towards MDA-MB-231 cells proliferation, which, taking into account the results presented in the PCA loading plot (Figure 5A), may be related to the high antioxidant activity (especially FRAP) of extracts from these species. However, this issue requires further studies. The remaining *Fabaceae* species, characterized by weaker activity against the MCF7 cell line in comparison to cluster B and the lowest antioxidant activity were classified into cluster C.

Although the classification of species based on the activity of extracts and the sum of isoflavones does not reflect the division of the analyzed *Fabaceae* representatives based on the content of individual isoflavones (Figure 2), it should be noted that a common feature of all species within cluster B was the simultaneous presence of genistin and ononin.

## 3. Materials and Methods

### 3.1. Chemical and Solutions

HPLC grade acetonitrile, water, and formic acid were purchased from Merck (Darmstadt, Germany). Reference standards were used for HPLC analysis of isoflavones: biochanin A, formononetin, genistein, genistein, glycitein, daidzein, daidzin, and ononin were purchased from Fluka Chemie (Buchs, Switzerland); sissotrin, prunetin, and puerarin were purchased from Extrasynthese (Lyone, France); and calycosin was purchased from Merck (Darmstadt, Germany). Methanol was from Avantor Performance Materials Poland S.A. (Gliwice, Poland). All cell lines, media, and supplements were purchased in Merck (Darmstadt, Germany).

### 3.2. Extraction Optimization—Design of the Experiment

To optimize the extraction procedure, two extraction techniques were compared, namely heat-reflux extraction (HRE) and ultrasound-assisted extraction (UAE). Within each technique, three parameters were considered: (1) the time of extraction, (2) the solvent concentration used, and (3) the ratio of plant material to solvent volume. In order to investigate the impact of the experimental parameters on the effectiveness of extraction, an experimental planning method was used, as described previously [55], namely the fractional factorial design of experiments: 3^3-1^, in which 1/3 of the full 3^3^ design was selected, with 9 different combinations of the chosen factors (parameters), resulting in 9 trials. The scheme of factor coding and the plan of the experiment with the original parameters and their levels are shown in Table 5.

### 3.3. Plant Material and Sample Preparation for Optimization Procedure

Samples of 0.2 g of the dried and ground *Trifolium pratense* herb were transferred into glass round-bottomed flasks and extracted by heat-reflux extraction, conducted on an LWT water bath (90 °C) (WSL, Świętochłowice, Poland), or ultrasonic-assisted extraction, conducted with ultrasonic bath Sonic 3 (Polsonic, Warsow, Poland) at room temperature. Each combination of the experimental parameters (one of nine) was tested in three replicates. The extraction was performed for 30, 60, and 120 min (HRE) and 10, 20, and 30 min (UAE). Methanol in three concentrations, 50%, 75%, and 100%, was used as the solvent. To investigate the effect of the plant material mass-to-solvent volume ratio on the extraction efficiency, three different ratios of 1:25 (0.2 g and 5 mL), 1:50 (0.2 g and 10 mL), and 1:125 (0.2 g and 25 mL) were used. After the extraction, the obtained extracts were filtered and transferred into 5 mL, 10 mL, or 25 mL volumetric flasks, and the isoflavones contents were analyzed by high-performance liquid chromatography (HPLC). Before the analysis, extracts were filtered through the 0.45 μm membrane filters into 1.5 mL vials.

### 3.4. HPLC Analysis Conditions

The qualitative and quantitative analyses of isoflavones in plant material were performed using the Dionex HPLC system (Dionex, Sunnyvale, CA, USA), equipped with a PDA 100 UV-VIS detector and a Hypersil Gold (C-18) column (5 μm, 250 × 4.6 mm). Analysis was carried out in gradient mode, with 1% formic acid in water (A) and acetonitrile (B), 5–60% B in 60 min, at a flow rate of 1 mL/min and the detection wavelengths 254 nm and 285 nm [56]. The compounds mentioned above were identified by comparing their retention times and UV spectra with those of the reference standards (calycosin, daidzin, daidzein, genistin, genistein, glycytein, formononetin, ononin, sissotrin, puerarin, prunetin). The isoflavones content was calculated by measuring the peak area with respect to the appropriate standard curve (concentration range 0.0625–1 mg/mL). All analyses were performed in three independent experiments, and the mean value was expressed in mg/g of d.m.

### 3.5. Plant Material for Quantitative Analysis

The above-ground parts of 32 species of plants from the *Fabaceace* family in full flowering stage were used. The plant material was collected in summer (June–August) 2024 from their natural habitats in the Lesser Poland province (Table 6). After collection, the species were identified by a botanist at the Department of Pharmacognosy, Jagiellonian University Medical College. The plants were dried in the dark at room temperature, then cut into smaller pieces, ground, and finally sieved. Voucher specimens (Table 6) were placed in the Department of Pharmacognosy Jagiellonian University Medical College.

### 3.6. Sample Preparation and Extraction for Quantitative Analysis

Samples of 0.2 g of ground plant material were transferred to glass round-bottom flasks. Plant material was extracted with 25 mL of 50% methanol for 60 min in a water bath (90 °C) under reflux. Obtained extracts were transferred to a volumetric flask of 25 mL. Isoflavones content was analyzed by high-performance liquid chromatography (HPLC), as described above. Before analyses, extracts were filtered through 0.45 μm membrane filters into 1.5 mL vials. All analysis were performed in 3 replicates.

### 3.7. Determination of Antioxidant Activity

The antioxidant activity was evaluated using the DPPH and FRAP assays, following the methodology previously described by Paśko et al. [57]. In the DPPH assay, 3.9 mL of a methanolic DPPH solution (25 mg/L) was combined with 0.1 mL of plant extract. The decrease in absorbance was monitored at 515 nm until it stabilized. Each sample was analyzed in triplicate, and the antioxidant capacity was reported as µM Trolox per gram of dry mass (d.m.). For the FRAP assay, a freshly prepared reagent (900 μL)—consisting of 2.5 mL of 10 mM TPTZ in 40 mM HCl, 2.5 mL of 20 mM FeCl_3_·H_2_O, and 25 mL of 0.3 M acetate buffer at pH 3.6—was mixed with 90 μL of distilled water and 30 μL of plant extract. The reaction mixture’s absorbance was then measured at 593 nm. All experiments were performed independently in triplicate, and the results were expressed as µM Fe^2+^/g d.m. Absorbance readings for both assays were obtained using a Biotek Synergy microplate reader (BioTek Instruments Inc., Winooski, VT, USA).

### 3.8. Determination of Cytotoxic and Antiproliferative Activity

Cytotoxic and antiproliferative activity was tested on human breast panel (ER-positive breast adenocarcinoma MCF7, ATCC HTB-22; ER-negative breast adenocarcinoma MDA-MB-231, ATCC HTB-26). Safety assessment was tested on human hepatoblastoma HepG2 cells and normal human thyroid Nthy-ori 3-1 and breast epithelial MCF10A cells. Cells were grown under standard conditions (37 °C, 5% CO2, relative humidity) and culture media (DMEM/F12 for MDA-MB-231 and HepG2, MEM with NEAA for MCF7, RPMI1640 for Nthy-ori 3–1, DMEM/F12 with 20 ng/mL epidermal growth factor (EGF), 10 μg/mL insulin, 0.5 μg/mL hydrocortisone, 100 ng/mL cholera toxin for MCF10A) supplemented with 10% fetal bovine serum (FBS) or 5% donor horse serum for MCF10A and 1% antibiotics solution (10,000 U penicillin and 10 mg streptomycin/mL). The stock solutions of the examined extracts, prepared in DMSO, were then diluted in the culture medium to the working concentrations (from 0 to 500 μg/mL). Cell viability was determined after 48 h of incubation by MTT assay, as previously described [58]. Cell proliferation was determined after 48 and 72 h of incubation by crystal violet assay, as previously described [59]. To assess the safety of the extracts, an MTT assay was performed, and, additionally, the proliferation of normal thyroid cells was evaluated. The absorbance was measured at 490 nm in viability assay and at 570 nm in proliferation assay using a Biotek Synergy microplate reader (BioTek Instruments Inc., Winooski, VT, USA). Doxorubicin was used as a reference cytostatic. All analyses were performed in three independent experiments, and the results are expressed as % of control, untreated cells (mean ± SD).

### 3.9. Statistical Analysis

Software (Extreme Outlier, v.5.2) delivered by MP system Co. (Kraków, Poland), with an algorithm implemented by Shoemaker and based on a robust technique proposed by Tukey (Shoemaker, 1999), was used. For quantitative analysis, Levene’s test was used to assess the equality of variances in the compared groups. Since the variances were unequal in the analyzed cases, the Welch test was used to assess differences between groups, followed by the James–Howell post-hoc test. Principal component analysis (PCA) and hierarchical cluster analysis (HCA) were conducted using Statistica v.13.3 (TIBCO Software Inc., Palo Alto, CA, USA). Prior to developing the PCA model, Bartlett’s sphericity test and the Kaiser–Meyer–Olkin (KMO) index were assessed. In the PCA model, *Fabaceae* species served as the cases, with activities (antioxidant, viability (after 48 h incubation), proliferation (after 48 h incubation), and the isoflavone content as the parameters. The PCA model was based on the correlation matrix. Hierarchical cluster analysis (CA) utilized standardized data and was performed using Ward’s method and Euclidean distance, with clustering based on Mojena’s rule. Graphs were generated using Statistica v.13.3. Statistical analysis for antioxidant, cytotoxic, and antiproliferative activities was performed using one-way analysis of variance (ANOVA), followed by Tukey’s post-hoc test. All experiments were conducted three times, and data were presented as mean ± standard deviation (SD). A significance level of *p* < 0.05 was considered statistically significant.

## 4. Conclusions

The conducted study comprehensively assessed the phytochemical composition and biological activity of selected wild-growing *Fabaceae* species in Poland, with particular emphasis on isoflavones. A particularly novel aspect of our study was the comprehensive biological evaluation of the extracts, the results of which indicated their chemopreventive potential against breast cancer cells. The present study not only highlights the high pharmacological potential of some wild *Fabaceae* species but also emphasizes the importance of integrating phytochemical profiling with bioactivity assays to identify promising candidates for chemopreventive and therapeutic applications. While *Trifolium pratense* and *Trifolium medium* remain well-known sources of isoflavones, species such as *Genista tinctoria* and *Ononis arvensis* deserve more scientific attention due to their potent biological activity and favorable safety profiles. Further studies should include fractionation and identification of active compounds, mechanistic studies on molecular targets, and in vivo models to assess efficacy in other hormone-dependent cancers as well as safety.

## Figures and Tables

**Table 1:** Quantitative results of isoflavones content in *Trifolium pratense*, based on the optimization of the extraction conditions, derived from experiments with a fractional factorial design.

Isoflavones Sum Content (mg/g d.m.), Mean ± SD			
Set Number	Content	Set Number	Content
1 UAE	3.62 ± 0.01 ^b^	1 HRE	4.53 ± 0.10 ^b^
2 UAE	7.40 ± 0.13 ^a^	2 HRE	10.09 ± 0.22 ^b^
3 UAE	5.13 ± 0.2 ^b^	3 HRE	7.67 ± 0.04 ^b^
4 UAE	7.96 ± 0.19 ^a^	4 HRE	12.56 ± 0.67 ^a^
5 UAE	5.76 ± 0.03 ^b^	5 HRE	8.35 ± 0.11 ^b^
6 UAE	3.63 ± 0.11 ^b^	6 HRE	5.54 ± 0.22 ^b^
7 UAE	4.89 ± 0.02 ^b^	7 HRE	8.75 ± 0.17 ^b^
8 UAE	4.51 ± 0.08 ^b^	8 HRE	5.67 ± 0.23 ^b^
9 UAE	7.53 ± 0.14 ^a^	9 HRE	13.27 ± 0.30 ^a^

UAE: ultrasound-assisted extraction, HRE: heat-reflux extraction, SD: standard deviation. Within each column, values that differ significantly (*p* < 0.05) from the highest value are marked with different superscript letters. The set number abbreviations are explained along with the parameter values and experimental conditions in Section 3.2.

**Table 2:** Comparison of the contents (mg/g d.m.) of individual isoflavones in *Trifolium pratense* and *Trifolium medium* (mean ± SD; n = 3).

Isoflavone	*Trifolium medium*	*Trifolium pratense*
biochanin A	1.69 ± 0.23 ^a^	0.17 ± 0.02 ^b^
daidzin	0.67 ± 0.03 ^a^	3.00 ± 0.05 ^b^
formononetin	n.d.	0.13 ± 0.02
genistein	0.07 ± 0.01	n.d.
genistin	1.97 ± 0.05 ^a^	1.05 ± 0.05 ^b^
ononin	11.84 ± 0.19 ^a^	6.85 ± 0.52 ^b^
sissotrin	10.45 ± 0.35 ^a^	1.35 ± 0.02 ^b^

n.d.—not determined. Within each row, values that differ significantly (*p* < 0.05) between species are marked with different superscript letters.

**Table 3:** The antioxidant capacities of the extracts from *Fabaceae* family (mean value ± SD; n = 3; results within each column marked with the same lowercase letter indicate no significant differences between the extract with the highest result; abbreviations: DPPH—1,1-diphenyl-2-picrylhydrazyl; FRAP—ferric reducing antioxidant power; TEAC—Trolox equivalent antioxidant capacity).

Species	DPPH (μM TEAC/g d.m.)	FRAP (μM/Fe^2+^/g d.m.)
*Cytisus scoparius*	178.1 ± 5.9 ^a^	232.2 ± 3.7 ^a^
*Genista tinctoria*	67.5 ± 2.3 ^b^	112.7 ± 1.7 ^b^
*Medicago x varia*	24.0 ± 0.7 ^b^	61.2 ± 3.6 ^b^
*Melilotus albus*	36.3 ± 4.6 ^b^	77.0 ± 9.9 ^b^
*Ononis arvensis*	73.3 ± 42.5 ^b^	143.2 ± 4.3 ^b^
*Trifolium arvense*	182.4 ± 9.3 ^a^	248.8 ± 8.6 ^a^
*Trifolium fragiferum*	30.4 ± 5.1 ^b^	72.5 ± 7.5 ^b^
*Trifolium incarnatum*	43.7 ± 6.1 ^b^	80.2 ± 9.1 ^b^
*Trifolium medium*	66.2 ± 5.2 ^b^	137.4 ± 7.7 ^b^
*Trifolium pratense*	103. ± 26.6 ^b^	189.6 ± 32.4 ^b^
*Trifolium resupinatum*	41.9 ± 4.7 ^b^	93.3 ± 9.6 ^b^
*Vicia grandiflora*	59.2 ± 5.0 ^b^	110.0 ± 6.0 ^b^

Within each column, values that differ significantly (*p* < 0.05) from the highest value are marked with different superscript letters.

**Table 4:** Basic parameters of the PCA model.

Eigenvalues (Cumulative)	% Total Variance (Cumulative (%))	Variables	Factor Loadings PC1	Factor Loadings PC2
2.92	41.78%	FRAP	0.775	−0.536
2.25	32.13%	DPPH	0.707	−0.616
(5.17)	(73.91%)	MCF7 viability	−0.561	−0.687
		MDA-MB-231 viability	−0.446	0.437
		MCF7 proliferation	−0.579	−0.736
		MDA-MB-231 proliferation	−0.789	0.151
		Isoflavones sum	0.594	0.596

**Table 5:** Optimization of isoflavones extraction conditions, for *Trifolium pratense*, using experiments with a fractional factorial design: parameter values and coded sets of experimental conditions.

	Time of Extraction (HRE, UAE: min)		Solvent Concentration (%)		Ratio of Plant Material to Solvent Volume	
Set Number	Parameter Value	Code	Parameter Value	Code	Parameter Value	Code
1	HRE: 30 UAE: 10	−1	50	−1	1:25	−1
2	HRE: 30 UAE: 10	−1	75	0	1:125	1
3	HRE: 30 UAE: 10	−1	100	1	1:50	0
4	HRE: 60 UAE: 20	0	50	−1	1:125	1
5	HRE: 60 UAE: 20	0	75	0	1:50	0
6	HRE: 60 UAE: 20	0	100	1	1:25	−1
7	HRE: 120 UAE: 30	1	50	−1	1:50	0
8	HRE: 120 UAE: 30	1	75	0	1:25	−1
9	HRE: 120 UAE: 30	1	100	1	1:125	1

HRE: heat-reflux extraction; UAE: ultrasound-assisted extraction.

**Table 6:** The list of plant species, included in the study, with the localization and geographical coordinates of their collection places.

Species	Localization	Geographical Coordinates	Voucher Specimens
*Anthyllis vulneraria*	Kraków	50.06′ N, 19.92′ E	KFg/2024/Av
*Astragalus cicer*	Kraków	50.08′ N, 20.03′ E	KFg/2024/Ac
*Coronilla varia*	Olkusz	50.31′ N, 19.57′ E	KFg/2024/Cv
*Cytisus scoparius*	Węgrzce Wielkie	50.02′ N, 20.12′ E	KFg/2024/Cs
*Genista tinctoria*	Kraków	50.03′ N, 19.91′ E	KFg/2024/Gt
*Lathyrus latifolius*	Wieliczka	49.99′ N, 20.04′ E	KFg/2024/Lal
*Lathyrus odoratus*	Gdów	49.90′ N, 20.19′ E	KFg/2024/Lo
*Lathyrus pratensis*	Kraków	50.06′ N, 19.92′ E	KFg/2024/Lpr
*Lotus corniculatus*	Olkusz	50.31′ N, 19.57′ E	KFg/2024/Lc
*Lupinus polyphyllus*	Olkusz	50.30′ N, 19.57′ E	KFg/2024/Lpo
*Medicago falcata*	Kraków	50.09′ N, 19.90′ E	KFg/2024/Mf
*Medicago lupulina*	Wieliczka	49.98′ N, 20.04′ E	KFg/2024/Ml
*Medicago x varia*	Kraków	50.06′ N, 19.95′ E	KFg/2024/Mv
*Melilotus albus*	Kraków	50.01′ N, 20.00′ E	KFg/2024/Ma
*Melilotus officinalis*	Kraków	50.06′ N, 19.95′ E	KFg/2024/Mo
*Onobrychis viciifolia*	Kraków	50.04′ N, 19.96′ E	KFg/2024/Ov
*Ononis arvense*	Kraków	50.03′ N, 19.91′ E	KFg/2024/Oa
*Trifolium arvense*	Gdów	49.90′ N, 20.18′ E	KFg/2024/Ta
*Trifolium campestre*	Wieliczka	49.98′ N, 20.03′ E	KFg/2024/Tc
*Trifolium fragiferum*	Kraków	50.02′ N, 19.97′ E	KFg/2024/Tf
*Trifolium hybridum*	Kraków	50.04′ N, 19.96′ E	KFg/2024/Th
*Trifolium incarnatum*	Łazany	49.95′ N, 20.16′ E	KFg/2024/Ti
*Trifolium medium*	Kraków	50.03′ N, 19.90′ E	KFg/2024/Tme
*Trifolium montanum*	Kraków	50.04′ N, 19.91′ E	KFg/2024/Tmo
*Trifolium pratense*	Kraków	50.01′ N, 20.00′ E	KFg/2024/Tp
*Trifolium repens*	Kraków	50.01′ N, 20.00′ E	KFg/2024/Trep
*Trifolium resupinatum*	Gdów	49.90′ N, 20.19′ E	KFg/2024/Tres
*Vicia angustifolia*	Kraków	50.08′ N, 19.92′ E	KFg/2024/Va
*Vicia grandiflora*	Kraków	50.07′ N, 19.87′ E	KFg/2024/Vg
*Vicia hirsuta*	Kraków	50.06′ N, 19.91′ E	KFg/2024/Vh
*Vicia sepium*	Kraków	50.07′ N, 19.87′ E	KFg/2024/Vs
*Vicia villosa*	Kokotów	50.01′ N, 20.07′ E	KFg/2024/Vv

## Data Availability

Date are contained within the article and Supplementary Material.

## Supplementary Materials

The following supporting information can be downloaded at: https://www.mdpi.com/article/10.3390/molecules30112379/s1, Table S1: Analytical parameters of the quantitative HPLC method for determination of isoflavones; Figure S1: Chromatograms and UV spectra of the isoflavones standards used in the study.
