# Supplementary material for: Quantitative Analysis of Isoflavones from Fabaceae Species and Their Chemopreventive Potential on Breast Cancer Cells
Source: Molecules. 2025 May 29;30(11):2379. doi: 10.3390/molecules30112379 (PMC12155718; doi:10.3390/molecules30112379)

Quantitative Analysis of Isoflavones from Fabaceae Species and Their Chemopreventive Potential on Breast Cancer Cells

Wojciech Pa´zdziora [1,2], Karolina Grabowska [2], Paweł Zagrodzki [3], Paweł Pa´sko [3], Ewelina Prochownik [3], Irma Podolak [2] and Agnieszka Galanty [2,*]

[1] Doctoral School of Medical and Health Sciences, Jagiellonian University Medical College, 16 Łazarza Str., 31-530 Cracow, Poland; wojciech.pazdziora@doctoral.uj.edu.pl
[2] Department of Pharmacognosy, Jagiellonian University Medical College, Medyczna 9, 30-688 Kraków, Poland; karolina1.grabowska@uj.edu.pl (K.G.); irma.podolak@uj.edu.pl (I.P.)
[3] Department of Food Chemistry and Nutrition, Jagiellonian University Medical College, Medyczna 9, 30-688 Kraków, Poland; pawel.zagrodzki@uj.edu.pl (P.Z.); p.pasko@uj.edu.pl (P.P.); ewelina.gajdzik@uj.edu.pl (E.P.)
* Correspondence: agnieszka.galanty@uj.edu.pl

Supplementary data

Table S1. Analytical parameters of the quantitative HPLC method for determination of isoflavones.

Parameter	biochanin A	calycosin	daidzin	formononetin	genistein	genistin	ononin	sissotrin
Retention time [min]	41.48	26.38	16.29	34.79	30.93	19.05	24.33	29.04
Slope	636.68	789.71	470.68	602.24	694.61	389.85	214.83	440.64
Intercept	20.12	13.23	-3.83	26.25	25.70	7.70	5.86	27.96
Regresion coefficient (R^2)	0.9961	0.9966	0.9974	0.9956	0.9958	0.9973	0.9896	0.9899
Linearity range [mg/ml]	0.0625 - 1.0	0.0625 -1.0	0.0625 - 1.0	0.0625 – 1.0	0.0625 – 1.0	0.0625 - 1.0	0.01 - 0.4	0.0625 - 1.0
LOD[1] [mg/mL]	0.079	0.091	0.024	0.079	0.089	0.040	0.022	0.123
LOQ[1] [mg/mL]	0.239	0.275	0.072	0.240	0.269	0.121	0.066	0.374
Intra-day RSD[2] [%]	1.12	1.81	1.60	1.98	1.34	1.59	1.95	1.89
Inter-day RSD[2] [%]	2.52	2.74	2.80	2.77	1.44	2.01	2.87	2.79

[1]LOD and LOQ: were calculated based on the standard error of residuals (Se) and the slope (a) of the calibration plots. LOD is the concentration estimated for the response equal to 10 Se, and LOQ is the concentration estimated for the response equal to 3.3 Se; [2]The accuracy and repeatability of the method were assessed by sixfold analysis of the concentration level 0.25 mg/mL of isoflavones. The same protocol was followed for three subsequent different days to study the intermediate precision of the proposed method.

Figure S1. Chromatograms and UV spectra of the isoflavones standards used in the study.

Biochanin A

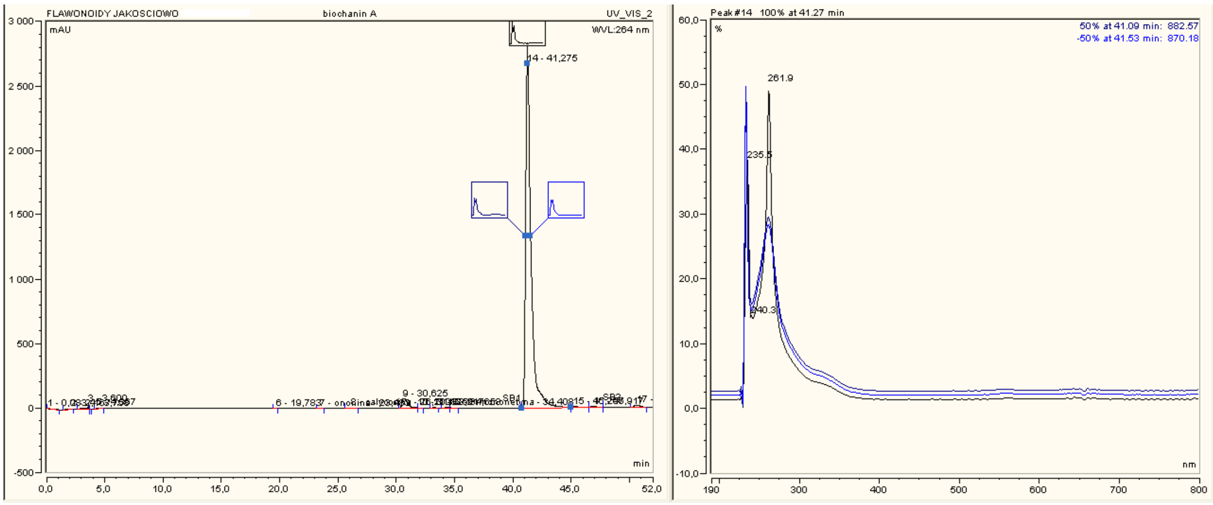

Calycosin

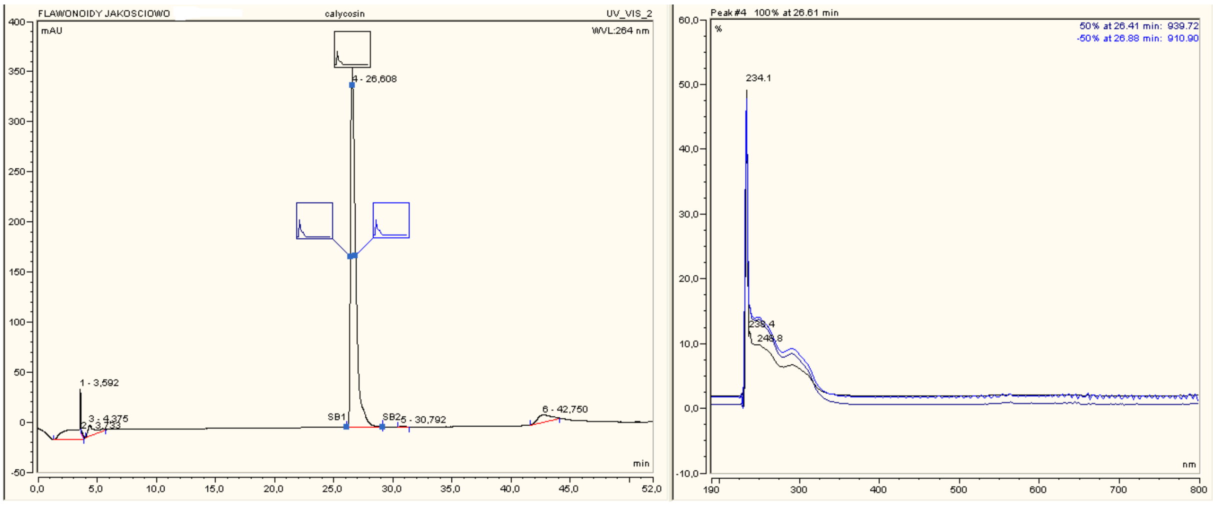

Daidzin

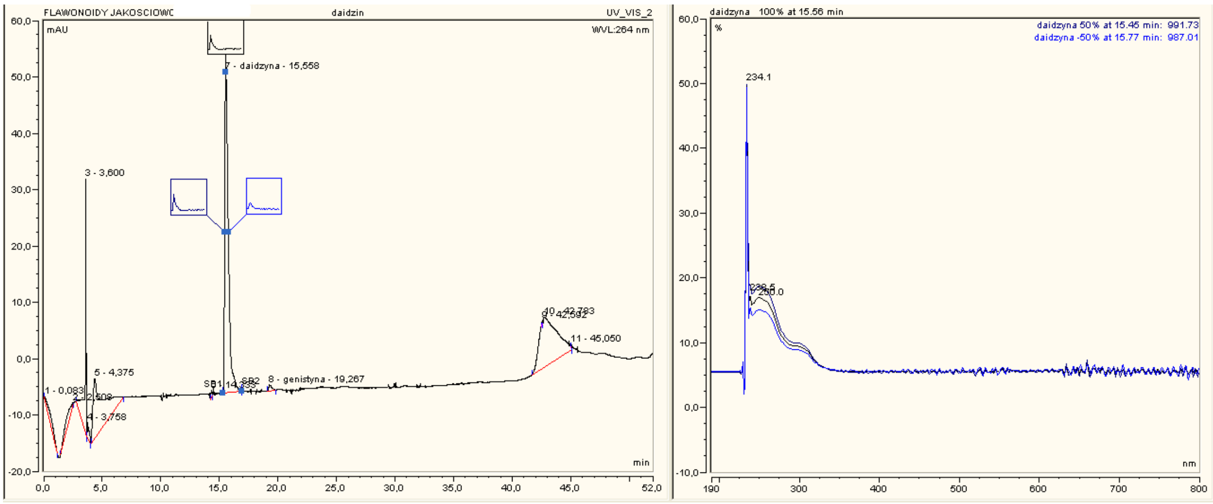

Formononetin

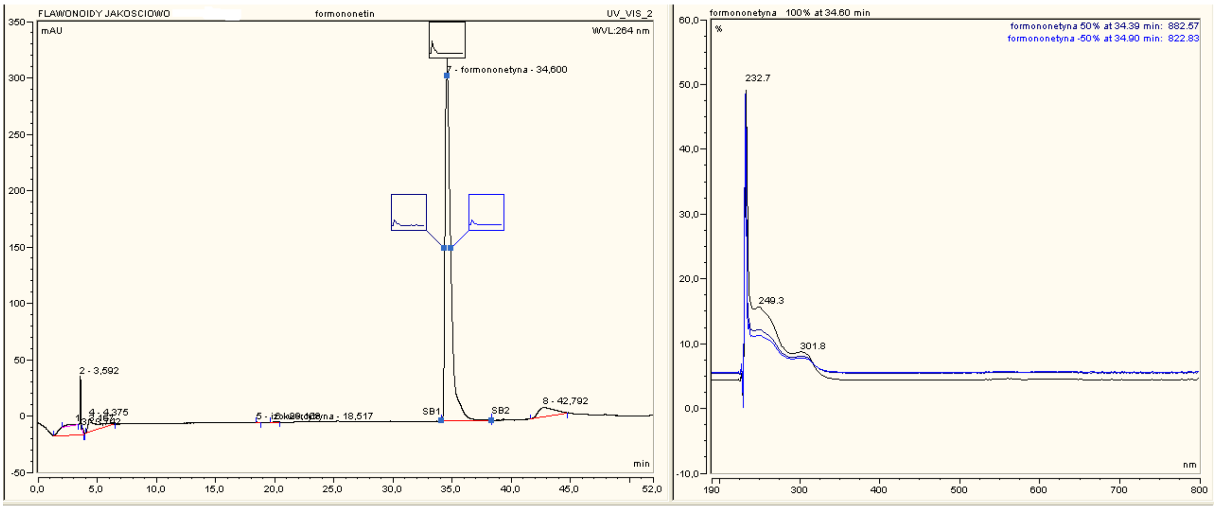

Genistin

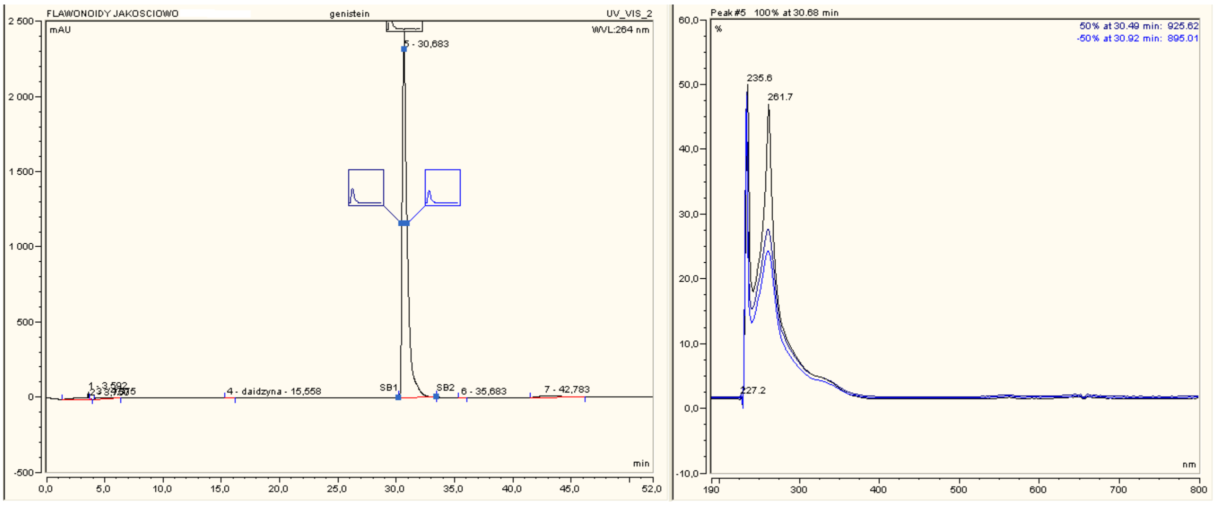

Sissotrin

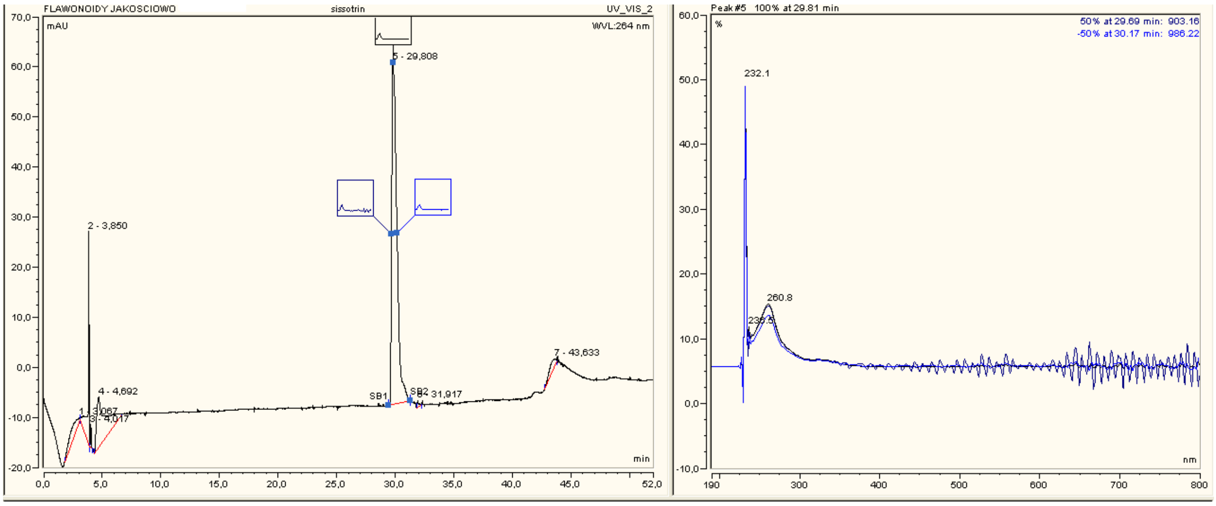

Supplement: Supplementary file 1 [file molecules-30-02379-s001.zip › molecules-3644527-supplementary.pdf]
